# Glacial refugia and the prediction of future habitat coverage of the South American lichen species *Ochrolechia austroamericana*

**DOI:** 10.1038/srep38779

**Published:** 2016-12-08

**Authors:** Martin Kukwa, Marta Kolanowska

**Affiliations:** 1 Department of Plant Taxonomy and Nature Conservation, The University of Gdańsk, Wita Stwosza 59, 80-308 Gdańsk, Poland; 2 Department of Biodiversity Research, Global Change Research Institute AS CR, Bělidla 4a. 603 00 Brno, Czech Republic

## Abstract

The biogeographic history of lichenized fungi remains unrevealed because those organisms rarely fossilize due to their delicate, often tiny and quickly rotting thalli. Also the ecology and factors limiting occurrence of numerous taxa, especially those restricted in their distribution to tropical areas are poorly recognized. The aim of this study was to determine localization of glacial refugia of South American *Ochrolechia austroamericana* and to estimate the future changes in the coverage of its habitats using ecological niche modeling tools. The general glacial potential range of the studied species was wider than it is nowadays and its niches coverage decreased by almost 25% since last glacial maximum. The refugial areas were covered by cool and dry grasslands and scrubs and suitable niches in South America were located near the glacier limit. According to our analyses the further climate changes will not significantly influence the distribution of the suitable niches of *O. austroamericana.*

Lichens (lichenized fungi) are an artificial group of various, distantly related fungal lineages, that share the dependence on photoautotrophic organisms^1^,^2^. They occur in almost all terrestrial ecosystems from cold Arctic and Antarctic vegetation to the communities of dry deserts and humid tropical rain forests^3^. Lichens are very sensitive to environmental and climate changes, caused by natural factors as well as human impact, especially to the increase (or rarely decrease) of air pollution^4^,^5^,^6^,^7^,^8^,^9^,^10^,^11^,^12^. Their distribution ranges changed during last several thousand years and were most probably connected with the postglacial history of plant communities^13^ and, as it is proven for plant species^14^,^15^,^16^,^17^ that they must have survived in the refugial areas, but there are no paleomycological data confirming that as lichens rarely fossilize due to their delicate, often tiny and quickly rotting thalli. Some lichen fossils are known e.g. from the Miocene deposits from California^18^ and amber, mostly from the Baltic sea^19^,^20^,^21^ but almost no records are known from the period of last glacial period as lichen thalli have been only very rarely preserved in mires or sediments^22^. Therefore, the only possibility to know the history and the past distribution of species in the last few thousand years the potential niche modeling appears as the most appropriate method.

During ongoing studies on lichenized and lichenicolous fungi of Bolivia, unexpectedly an interesting species, *Ochrolechia austroamericana* (Räsänen) Räsänen, was found in this country and also Ecuador^23^. Until very recently this lichen was known exclusively from southern South America from Argentina, Chile and Uruguay^24^,^25^ but the new findings extended its distribution much more northward. At present the species can be considered as widely distributed in South America, though not many records are known. Therefore, as numerous regions of South America, especially the highest parts of the Andes, are still poorly explored in terms of lichen occurrences, the new data brought us to a question how the potential range of *O. austroamericana* may look like in this continent. For that reason we decided to use the ecological niche modeling (ENM) tools to estimate the distribution of the suitable habitats of this species. These tools also allowed identifying the locations of possible refugia of this species during the last glacial maximum (LGM, from 26500 to 19000–20000 years ago) as well as to predict the further changes of its potential range due to the climate change and drying of the Andes.

In lichenology the ENM tools were used so far in the taxonomic research on *Fuscopannaria confusa* (P.M. Jørg.) P.M. Jørg., a rare lichen restricted to very humid localities in boreal forests with already recognized ecology and habitat requirements^26^. Moreover, species distribution modeling methods were used in more or less local conservation planning^27^,^28^,^29^. Our work is the first one, which presents data on the potential distribution from the past to the future as well the first such dealing with lichenized fungi in South America. Because so far MaxEnt application was rarely used in lichen species distribution modeling the additional aim of the present study was to evaluate differences in the models created using this application with various input data.

## Results

### Ecological niche modeling evaluation

The area under the ROC curve (AUC) for each model created based on different dataset was calculated to estimate the trustworthiness of the analysis. AUC values were calculated by MaxEnt application automatically and it describes the probability that the model scores a random presence site higher than a random background site^30^,^31^ (Phillips *et al*.). All projected niche models received high AUC scores (Table 1) that indicates a high reliable performance of the analysis. Models were then projected onto the spatial layers representing the current, past and future climatic conditions.

The niche overlap test was also used to evaluate differences between models created with all available climatic and altitudinal data and those based on reduced “bioclims” dataset. The obtained results indicated some incongruities between the created models - for LGM simulation the niche overlap statistics were calculated as I = 0.899, D = 0.699 and for the present time I = 0.899, D = 0.621. The internal tests of GARP models showed high reliability of these analysis (Supplementary Table S1).

### Current potential range

**Figure 1:**
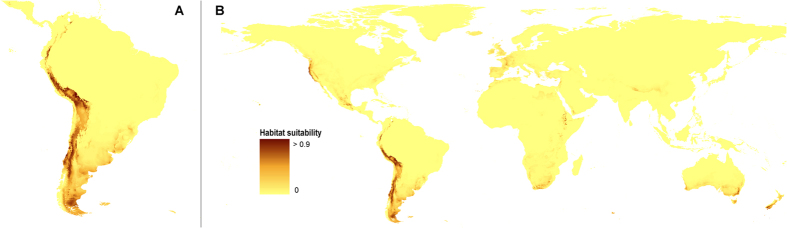
Current distribution of ecological niches of *O. austroamericana* in South America (**A**) and whole world (**B**) based on “All” model. Maps generated in ArcGis 9.2^78^ (http://www.esri.com/).

According to the most reliable model (Fig. 1), the niches of the studied species are distributed from Venezuelan Cordillera de Merida along the Andean range to southern Argentina with the highest concentration of the suitable habitats in Central Andes. Somewhat less appropriate conditions are found in the area of Altiplano. In Northern America the proper niches are found along the Pacific coast, west of Coast Ranges. Less suitable habitats are found along Sierra Nevada. In Mexico some potentially available niches are located in the southern part of Sierra Madre. Moreover, the model indicate the existence of suitable niches outside the Neotropics - in French Massif Central, Ethiopian Highlands, African Great Karoo as well as in southern New Zealand and Australian Great Dividing Range. Two other models which received lower AUC scores are presented in Supplementary Figure S2.

### Glacial refugia

**Figure 2:**
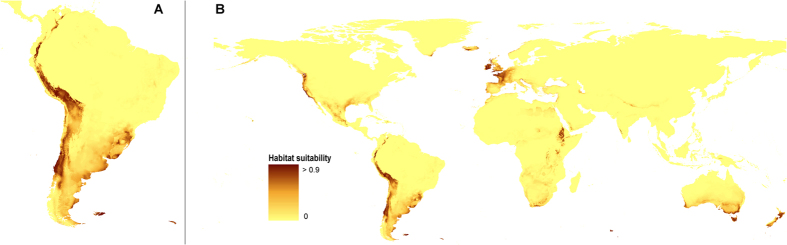
Distribution of ecological niches of *O. austroamericana* during LGM in South America (**A**) and whole world (**B**) based on “All” model. Maps generated in ArcGis 9.2^78^ (http://www.esri.com/).

The general distribution of the suitable niches of *O. austroamericana* in LGM in the most reliable “All” model is congruent with the present potential range of this species (Fig. 2). There are, however, several regions which current bioclimatic conditions are not appropriate for this lichen. In LGM potentially available habitats were located in Falkland Islands, Atlantic coastal regions of Argentina and Uruguay as well as in south-western Iceland and Ireland. The general glacial potential range of *O. austroamericana* was apparently wider than it is nowadays.

In South America the areas indicated in the models as potentially available for *O. austroamericana* were covered by cool and dry grasslands and scrubs^32^. Interestingly, the suitable niches in North America were located near the glacier limit.

Two other models which received lower AUC scores are presented in Supplementary Figure S3.

### Limiting factors and niches overlap

The geographical differences in the distribution of *O. austroamericana* niches during LGM and present time shown in the most reliable models were confirmed in the statistics of niche overlap: D = 0.641, I = 0.889 calculated for the most reliable datasets (“All”). In those models the main climatic factors limiting distribution of *O. austroamericana* are altitude and temperature constancy (bio3 and bio4; Table 2). The most optimal altitude for the species occurrence is about 4500 m.

In the models with reduced climatic and altitudinal datasets the temperature seems to be crucial for the studied species distribution. In “SelArea” models the most important variables contributing in the models were mean temperature of the wettest quarter (bio8) and annual mean temperature (bio1). The latter factor together with isothermality (bio3) were critical for “SelLay” models. The niche overlap statistics in “SelLay” is very similar to that calculated for the most reliable models (D = 0.629, I = 0.875), but when considering exclusively South America (“SelArea”) the niche overlap is higher receiving the values of D = 0.725 and I = 0.922.

### Further changes

**Figure 3:**
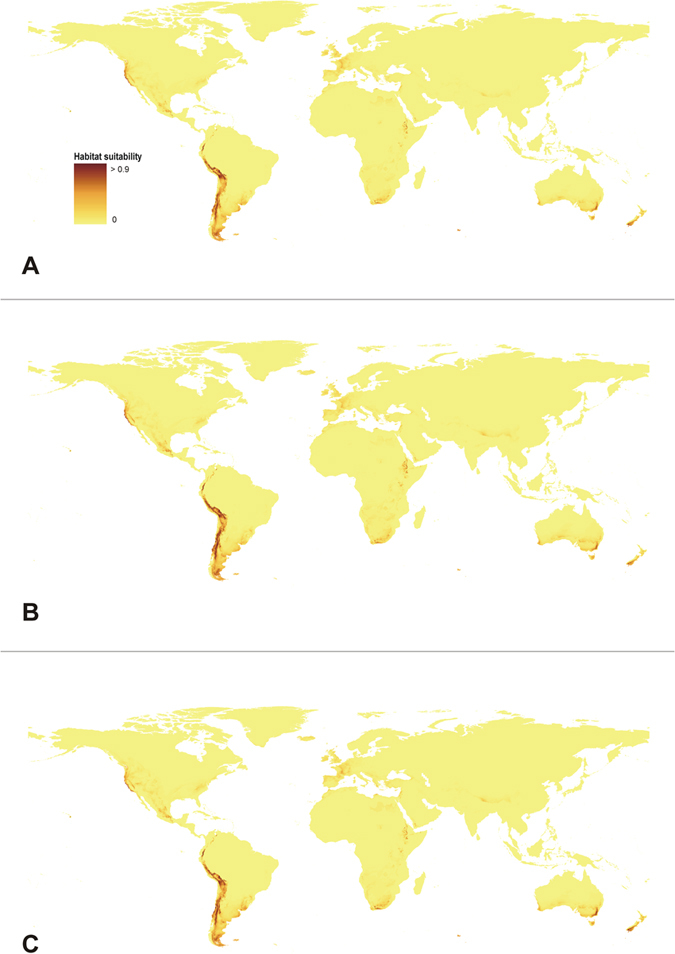
Predicted world-wide distribution of ecological niches of *O. austroamericana* in 2080 based on A1b (**A**) A2a (**B**) and B2a (**C**) climate changes scenarios based on “All” model. Maps generated in ArcGis 9.2^78^ (http://www.esri.com/).

**Figure 4:**
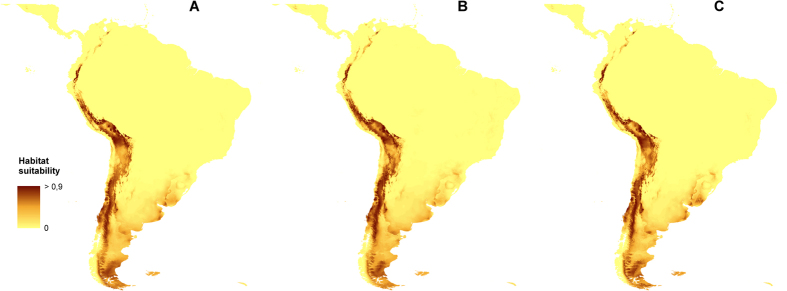
Predicted South American distribution of ecological niches of *O. austroamericana* in 2080 based on A1b (**A**) A2a (**B**) and B2a (**C**) climate changes scenarios based on “All” model. Maps generated in ArcGis 9.2^78^ (http://www.esri.com/).

Apparently, the further climate changes will not significantly influence the distribution of the suitable niches of *O. austroamericana.* The most reliable models created for three different climate change scenarios are presented in Figs 3 and 4. Two other models which received lower AUC scores are presented in Supplementary Figure S4.

The most insignificant global changes in the geographical distribution of the suitable niches in comparison to the present time will be observed in A2a scenario (Table 3) based on the conducted niche overlap test. The coverage of the most proper habitats, with the suitability above 0.7, will decrease in their surface in A1b and B2a models while their coverage in A2a scenario will increase in both analysed areas - whole World and South America only. The coverage of the areas characterized by the different suitability for the studied species is compiled in Tables 4 and 5. The similar tendency was observed based on GARP models analysis (Supplementary Figure S5, Supplementary Table S6).

## Discussion

Species distribution models have become increasingly noticeable in ecological and biogeographical research^33^. Mostly because ecologists need ways of rapidly assessing the impacts of climate change on large numbers of species for which the occurrence data are often the only source of information^34^. While several critical opinion on ENM analyses were presented in the last years^35^, MaxEnt seems to be the most reliable application for modeling species distribution. Its usefulness was also tested in case of rare organisms^36^,^37^. Noteworthy, some of the studies indicating inappreciable usefulness of ENM tools^38^ (Beale *et al*.) were called into question by the subsequent researchers^39^.

In our research we tested three approaches to evaluate the past, present and future distribution of the suitable niches of poorly known lichen species. Some of the previous studies indicated that the correlations between the environmental data used in the modeling should be reduced and that the correlated variables should be excluded from the analysis^40^. Also it was suggested that using restricted area in ENM analysis is more reliable than calculating habitat suitability in the global scale^41^. According to the received AUC scores the most reliable model was created based on all available climatic and altitudinal data and it was constructed for the whole globe. We therefore would postulate that all potentially useful climatic variables and altitudinal data should be used in ENM studies, especially when ecological information about the studied taxon are poor and they do not allow to discriminate any climatic data as irrelevant. Noteworthy, despite slight differences in the trustworthiness of the three conducted analyses all created models indicated similar areas that could be occupied by *Ochrolechia austroamericana.* In our opinion lichen species distribution modeling with MaxEnt may be extended into new fields and it would be especially useful in reconstructing their past distribution and potential migration routes. While future habitat loss of the lichens became emerging question in lichenology^42^,^43^, so far MaxEnt was not implemented in any of those studies. While the limitations of such predictions are well-known and their verification is not possible in the present time, we believe that the vulnerability of specific areas indicated in the modeling should be taken into consideration prior to planning conservation actions.

Despite *Ochrolechia austroamericana* has been reported from rather limited number of localities^23^,^24^, the ENM method has shown its potential distribution range can be much wider.

The analyses have shown the suitability of habitats on almost every continent; however its occurrence is highly improbable in most regions as the genus *Ochrolechia* A.Massal. was a subject of several taxonomic treatments over the past c. 30 years^24^,^25^,^44^,^45^,^46^,^47^,^48^ and *O. austroamericana* was never found outside South America. In general, most *Ochrolechia* species have distributions rather restricted to one continent or region, and that can be possibly related to the limited dispersal of diaspores (ascospores), which are relatively large^49^. Only very few species, e.g. the tropical *O. africana* Vain., are known to have wider range^25^,^45^ but if they truly represent one evolutionary lineage or several cryptic species, has not been settled yet. However, in the light of recent studies, which showed several lichenized fungi to represent numerous phylogenetically distinct clades^50^,^51^, we suspect this scenario to be more adequate in this case.

Concerning the most probable potential distribution range we consider that *O. austroamericana* occurs only South America from Venezuela to southern Argentina with, as our modeling has shown, the highest concentration of the suitable habitats in Central Andes. As it appears to be restricted to cooler climate conditions, it could not spread more northward due to the lack of appropriate spreading passages in Central America.

Apparently, the current distribution of the suitable habitats of *O. austroamericana* results from location of its glacial refugia and no long-distance dispersal of this lichen is observed. Its niches coverage decreased by almost 25% since LGM. Most of the loss is observed within the Pampas and in the high regions of Central and Southern Andes. We interpret the first issue as related with the coast line regression after Late Glacial that significantly affected local climatic conditions^52^. The loss of the habitats along the Andes may be caused by the warming of the high-Andean regions that was documented for numerous South American regions and which has been intensified in the last three decades, and, as the consequence, the vertical shift of vegetation belts in the altitudinal gradient, i.e. uppering the forest line^53^,^54^,^55^,^56^,^57^,^58^,^59^,^60^.

Surprisingly, the future distribution of *O. austroamericana* will not change much from the present range of the species. The difference between the three created models for 2080 differ in the suitability of the Atacama Desert and lower parts of the eastern slopes of Central Andes for the studied species occurrence. The studies of Boulanger *et al*.^61^ indicated that in twenty-first century the amplitude of the seasonal cycle will tend to increase in southern South America, while in northern South America the amplitude of the seasonal cycle would be reduced; that explains the relatively little changes in the general distribution of the species. However, while all emission paths tend to show the same pattern of warming, the highest warming is predicted in A2 storyline. This scenario is the only one which predicts increase of the suitable niches cover for *O. austroamericana.* We interpret it as a result of a tree-line location change^62^ as the lowering of the forest limit due to the climate change will reduce vegetation cover and may expose additional areas appropriate for *O. austroamericana*.

## Materials and Methods

**Figure 5:**
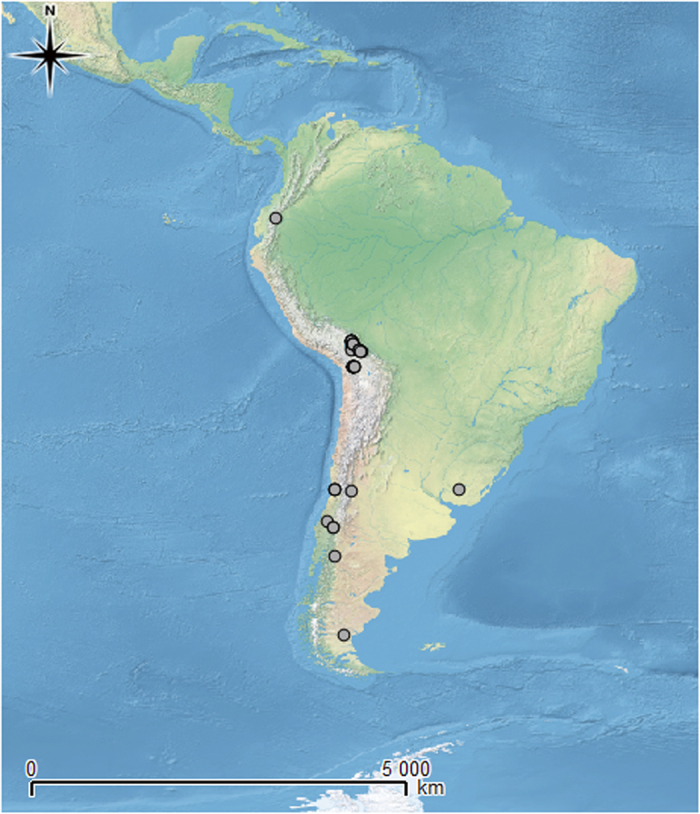
Localities of *O. austroamericana* in South America. Map generated in QGIS 2.2.0^79^ (http://hub.qgis.org/projects/quantum-gis).

The list of localities used in the modeling was compiled based on the examined samples exclusively. As the source of data we used specimens, including type material, from herbaria B, BM, GZU, H, KRAM, LPB, PRA, and UGDA, which were revised by the first author. All samples were investigated by thin-layer chromatography to detect diagnostic secondary lichen metabolites, crucial for the identification of *Ochrolechia* species. Methods were followed Orange *et al*.^63^. Most of those records have been already published by Messuti and Lumbsch^24^ and Kukwa *et al*.^23^ (data from Bolivia and Ecuador). Only the locations that could be precisely placed on the map were applied in the ENM analysis. The database of a total of 44 specimens was created from 21 different localities (Fig. 5). One specimen was excluded from the study as most probably it was mislabelled (the specimen was supposed to be collected in the middle of town in area without rock outcrops, the only substrate which *O. austroamericana* inhabits).

The ecological niche modeling was conducted using maximum entropy method implemented in MaxEnt version 3.3.2^18^,^64^,^65^ based on the species presence-only observations. In total, 20 different locations of *O. austroamericana* were used (Supplementary Table S7), which is more than the minimum number required to obtain reliable predictions in MaxEnt application^66^,^67^.

Three different approaches were used to conduct analysis. In the first one (“All”) as input data, all 19 climatic variables (“bioclims”, Table 6) in 2.5 arc minutes (±21.62 km^2^ at the equator) developed by Hijmans *et al*.^68^ were used together with the altitudinal data (Alt) and the models were created for the whole globe. From the second dataset (“SelLay”), we removed altitudinal data and seven “bioclims” due to their significant correlation (above 0.9) as evaluated by the Pearsons’ correlation coefficient calculation computed using ENMTools v1.3. The following variables were excluded from the dataset: alt, bio6, bio7, bio9, bio10, bio11, bio16 and bio17. This analysis was also made for the whole globe. The last set of models (“SelArea”) was made using the same, reduced number of variables and the area was restricted to longitude of 95°-30°W and latitude of 13°N-60°S.

In all analysis the maximum iterations was set to 10000 and convergence threshold to 0.00001. For each run, 15% of the data were used to be set aside as test points^69^. The “random seed” option which provided random test partition and background subset for each run was applied. The run was performed as a bootstrap with 1000 replicates, and the output was set to logistic. All operations on GIS data were carried out on ArcGis 9.3 (ESRI). The bioclimatic data for the LGM were developed and mapped by Paleoclimate Modeling Intercomparison Project Phase II^70^ based on an atmosphere-ocean coupled general circulation model (AOGCM).

To assess potential range of *O. austroamericana* in 2080 three different climate change scenarios were applied into modeling^71^,^72^. A1b (CCCMA-CGCM3 simulation), A2a (CCCMA-CGCM2 simulation) and B2a (CCCMA-CGCM2 simulation). A1b scenario is characterized by the balance across all energy sources (where balanced is defined as not relying too heavily on one particular energy source, on the assumption that similar improvement rates apply to all energy supply and end-use technologies). The A2 storyline describes a highly heterogeneous future world with regionally oriented economies. The main driving forces are a high rate of population growth, increased energy use, land-use changes and slow technological change. The B2 is scenario with a general evolution towards environmental protection and social equity. The datasets used in the analysis are available on CIAS website (http://ccafs-climate.org). The coverage of the suitable niches were calculated in order to measure further changes in the habitat availability for both whole world and South America only.

The corresponding analyses based on dataset that received the highest AUC scores using GARP algorithm applying the best subsets^73^ were conducted to verify MaxEnt models for the present and future time. These models were created using openModeller^74^.

The niche overlap tests implemented in ENMTools application were used to evaluate the overlap of the potential ranges modeled for LGM and present time as well as to estimate differences between tested models “All” and “SelLay”. The overlap was measured using Schoener’s D (D)^75^ and I statistic (I)^76^,^77^. Schoener’s D was developed initially to compare diet and microhabitats and here it is used with assumption that direct measures of local species density are compared with each other. The I statistic is basing on Hellinger distance and measures the ability of the model to estimate the true suitability of habitat. Both metrics range from 0 (niches are completely different) to 1 (overlap).

## Additional Information

**How to cite this article**: Kukwa, M. and Kolanowska, M. Glacial refugia and the prediction of future habitat coverage of the South American lichen species *Ochrolechia austroamericana*. *Sci. Rep.* **6**, 38779; doi: 10.1038/srep38779 (2016).

**Publisher's note:** Springer Nature remains neutral with regard to jurisdictional claims in published maps and institutional affiliations.

## Supplementary Material

Supplementary Dataset 1

## Figures and Tables

**Table 1:** The average training AUC for the replicate runs (standard deviation values given in parentheses) for various models and datasets.

Dataset	Present	LGM	A1b	A2a	B2a
All	0.998 (0.001)	0.998 (0.001)	0.997 (0.001)	0.997 (0.001)	0.997 (0.001)
SelArea	0.991 (0.004)	0.990 (0.004)	0.991 (0.004)	0.991 (0.004)	0.991 (0.004)
SelLay	0.997 (0.001)	0.997 (0.001)	0.997 (0.001)	0.997 (0.001)	0.997 (0.001)

**Table 2:** Estimates of relative contributions of the environmental variables to the Maxent models in various datasets.

Dataset	Model	Var_1	Var_2	Var_3	Var_4	Var_5
All	Present	alt (35.6)	bio3 (15.6)	bio4 (15)	bio11 (8.1)	bio1 (6.9)
	LGM	alt (38.9)	bio4 (15.3)	bio3 (13.4)	bio11 (9.4)	bio18 (4.9)
SelArea	Present	bio8 (38.9)	bio1 (14.6)	bio13 (10.5)	bio18 (7.5)	bio19 (7.0)
	LGM	bio8 (38.7)	bio1 (13.9)	bio13 (10.4)	bio18 (8.7)	bio19 (5.8)
SelLay	Present	bio3 (38.6)	bio1 (32.4)	bio4 (9.2)	bio8 (5.3)	bio18 & bio13 (4.5)
	LGM	bio3 (9.9)	bio1 (31.4)	bio4 (8)	bio8 (6.5)	bio18 (4.3)

**Table 3:** Overlap of *O. austroamericana* suitable niches between present time and various climate changes scenarios.

D \ I	Present time	A1b	A2a	B2a
Present time	x	0.984	0.988	0.983
A1b	0.877	x	0.983	0.990
A2a	0.891	0.871	x	0.986
B2a	0.871	0.894	0.881	x

**Table 4:** World-wide coverage of areas characterized by bioclimatic conditions of various suitability for *O. austroamericana* in present time and in various climate changes scenarios.

Suitability	Present time	A1b	A2a	B2a
>0.8	99689.82 km^2^	99754.68 km^2^	144940.48 km^2^	125958.23 km^2^
0.7–0.8	273622.72 km^2^	177543.44 km^2^	263158.64 km^2^	219075.46 km^2^
>0.7	373312, 54 km^2^	277298, 12 km^2^	408099, 12 km^2^	345033, 69 km^2^

**Table 5:** Coverage of areas characterized by the suitability of over 0.7 for *O. austroamericana* in South America in present time and in various climate changes scenarios.

LGM	Present time	A1b	A2a	B2a
36191, 88 km^2^	27284 km^2^	19349 km^2^	32170, 56 km^2^	23717, 14 km^2^

**Table 6:** Bioclimatic variables used in the ENM analysis.

Code	Variable
bio1	Annual mean temperature
bio2	Mean diurnal range = mean of monthly (max temp-min temp)
bio3	Isothermality (bio2/bio7) (*100)
bio4	Temperature seasonality (standard deviation *100)
bio5	Max temperature of warmest month
bio6	Min temperature of coldest month
bio7	Temperature annual range (bio5-bio6)
bio8	Mean temperature of wettest quarter
bio9	Mean temperature of driest quarter
bio10	Mean temperature of warmest quarter
bio11	Mean temperature of coldest quarter
bio12	Annual precipitation
bio13	Precipitation of wettest month
bio14	Precipitation of driest month
bio15	Precipitation seasonality (coefficient of variation)
bio16	Precipitation of wettest quarter
bio17	Precipitation of driest quarter
bio18	Precipitation of warmest quarter
bio19	Precipitation of coldest quarter

## References

[b1] Sipman H. J. M. & Aptroot A. Where are the missing lichens? Mycol. Res. 105, 1433–1439 (2001).

[b2] Miądlikowska J. . A multigene phylogenetic synthesis for the class Lecanoromycetes (Ascomycota): 1307 fungi representing 1139 infrageneric taxa, 317 genera and 66 families. Mol. Phylogenet. Evol. 79, 132–168 (2014). 24747130 10.1016/j.ympev.2014.04.003 PMC4185256

[b3] Nash T. H. Lichen Biology, 2nd ed. (Cambridge University Press, 2008).

[b4] van Dobben H. F. Decline and recovery of epiphytic lichens in an agricultural area in the Netherlands (1900-1988). Nova Hedwigia 62, 477–485 (1996).

[b5] Rusu A.-M., Jones G. C., Chimonides P. D. J. & Purvis W. O. Biomonitoring using the lichen *Hypogymnia physodes* and bark samples near Zlatna, Romania immediately following closure of a copper ore-processing plant. Environ. Pollut. 143, 81–88 (2006). 16368174 10.1016/j.envpol.2005.11.002

[b6] Wolseley P., James P. W., Theobald M. R. & Sutton M. A. Detecting changes in epiphytic lichen communities at sites affected by atmospheric ammonia from agricultural sources. Lichenologist 38, 161–176 (2006).

[b7] Motiejūnaitė J. Epiphytic lichen community dynamics in deciduous forests around a phosphorus fertiliser factory in central Lithuania. Environ. Pollut. 146, 341–349 (2007). 16725244 10.1016/j.envpol.2006.03.034

[b8] Sparrius L. B. Response of epiphytic lichen communities to decreasing ammonia air concentrations in a moderately polluted area of the Netherlands. Environ. Pollut. 146, 375–379 (2007). 16714078 10.1016/j.envpol.2006.03.045

[b9] Otnyukova T. & Sekretenko O. P. Spatial distribution of lichens on twigs in remote Siberian silver fir forests indicates changing atmospheric conditions. Lichenologist 40, 243–256 (2008).

[b10] van Herk C. M. Climate change and ammonia from cars as notable recent factors influencing epiphytic lichens in Zeeland, Netherlands. Bibl. Lichenol. 99, 205–224 (2009).

[b11] Olsen H. B., Berthelsen K., Andersen H. V. & Søchting U. *Xanthoria parietina* as a monitor of ground-level ambient ammonia concentrations. Environ. Pollut. 158, 455–461 (2010). 19781828 10.1016/j.envpol.2009.08.025

[b12] Żółkoś K., Kukwa M. & Afranowicz-Cieślak R. Changes in the epiphytic lichen biota in Scots pine (*Pinus sylvestris*) stands affected by a colony of grey heron (*Ardea cinerea*): a case study from northern Poland. Lichenologist 45, 815–823 (2013).

[b13] Printzen C. & Lumbsch H. T. Molecular evidence for the diversification of extant lichens in the Late Cretaceous and Tertiary. Mol. Phylogenet. Evol. 17, 379–387 (2000). 11133192 10.1006/mpev.2000.0856

[b14] Colyn M., Gautier-Hion A. & Verhaven W. A re-appraisal of palaeoenvironmental history in central Africa: evidence for a major fluvial refuge in the Zaire Basin. J. Biogeogr. 18, 403–407 (1991).

[b15] Segovia R. A., Pérez M. F. & Hinojosa L. F. Genetic evidence for glacial refugia of the temperate tree *Eucryphia cordifolia* (Cunoniaceae) in southern South America. Am. J. Bot. 99, 121–129 (2012). 22210838 10.3732/ajb.1100013

[b16] Tzedakisemail P. C., Emerson B. C. & Hewitt G. M. Cryptic or mystic? Glacial tree refugia in northern Europe. Trends Ecol. Evol. 28, 696–704 (2013). 24091207 10.1016/j.tree.2013.09.001

[b17] Juřičková L., Horáčková J. & Ložek V. Direct evidence of central European forest refugia during the last glacial period based on mollusc fossils. Quat. Res. 82, 222–228 (2014).

[b18] Peterson E. B. An overlooked fossil lichen (Lobariaceae). Lichenologist 32, 298–300 (2000).

[b19] Poinar G. O. Jr. Peterson E. B. & Platt J. L. Fossil *Parmelia* in New World amber. Lichenologist 32, 263–269 (2000).

[b20] Rikkinen J. & Poinar G. O. Jr. Fossilised *Anzia* (Lecanorales, lichen-forming Ascomycota) from European tertiary amber. Mycol. Res. 106, 984–990 (2002).

[b21] Rikkinen J. & Poinar G. O. Jr. A new species of *Phyllopsora* (Lecanorales, lichen-forming Ascomycota) from Dominican amber, with remarks on the fossil history of lichens. J. Exp. Bot. 59, 1007–1011 (2008). 18319239 10.1093/jxb/ern004

[b22] Knaap van der W. O., Aptroot A. & Oosterveld P. A 7500-year-old record of *Peltigera aphthosa* from Spitsbergen. Lichenologist 21, 90–91 (1989).

[b23] Kukwa M., Rodriguez Flakus P. & Flakus A. Notes on the lichen genus *Ochrolechia* in Bolivia. Polish Bot. J. 58, 691–695 (2013).

[b24] Messuti M. I. & Lumbsch H. T. A revision of the genus *Ochrolechia* in southern South America. Bibl. Lichenol. 75, 33–46 (2000).

[b25] Kukwa M. The lichen genus Ochrolechia in Europe (Fundacja Rozwoju Uniwersytetu Gdańskiego, 2011).

[b26] Carlsen T. . Species delimitation, bioclimatic range, and conservation status of the threatened lichen *Fuscopannaria confusa*. Lichenologist 44, 565–575 (2012).

[b27] Martínez I., Carreño F., Escudero A. & Rubio A. Are threatened lichen species well-protected in Spain? Effectiveness of a protected areas network. Biol. Cons. 133, 500–511 (2006).

[b28] Binder M. D. & Ellis C. J. Conservation of the rare British lichen *Vulpicida pinastri*: changing climate, habitat loss and strategies for mitigation. Lichenologist 40, 63–79 (2008).

[b29] Cameron R. P., Neily T. & Clayden S. R. Distribution prediction model for *Erioderma mollissimum* in Atlantic Canada. Bryologist 114, 231–238 (2011).

[b30] Phillips S. J., Anderson R. & Schapire R. E. Maximum entropy modeling of species geographic distributions. Ecol. Modell. 190, 231–259 (2006).

[b31] Phillips S. J. . Sample selection bias and presence-only distribution models: implications for background and pseudo-absence data. Ecol. Appl. 19, 181–197 (2009). 19323182 10.1890/07-2153.1

[b32] Olson J. S., Watts J. A. & Allison L. J. Carbon in live vegetation of major world ecosystems (Oak Ridge National Laboratory, 1983).

[b33] Guisan A. & Zimmermann N. E. Predictive habitat distribution models in ecology. Ecol. Modell. 135, 147–186 (2000).

[b34] Araújo M. B. & Rahbek C. How does climate change affect biodiversity? Science 313, 1396–1397 (2006). 16959994 10.1126/science.1131758

[b35] Chapman D. S. Weak climatic associations among British plant distributions. Global Ecol Biogeogr 19, 831–841 (2010).

[b36] Kumar S. & Stohlgren T. J. Maxent modeling for predicting suitable habitat for threatened and endangered tree *Canacomyrica monticola* in New Caledonia. J. Ecol. Nat. Environ. 1, 94–98 (2009).

[b37] Rebelo H. & Jones G. Ground validation of presence-only modelling with rare species: a case study on barbastelles *Barbastella barbastellus* (Chiroptera: Vespertilionidae). J. Appl. Ecol. 47, 410–414 (2010).

[b38] Beale C. M., Lennon J. J. & Gimona A. Opening the climate envelope reveals no macroscale associations with climate in European birds. Proc. Natl. Acad. Sci. USA 105, 14908–14912 (2008). 18815364 10.1073/pnas.0803506105 PMC2567466

[b39] Araújo M. B., Thuiller W. & Yoccoz N. G. Reopening the climate envelope reveals macroscale associations with climate in European birds. Proc. Natl. Acad. Sci. USA 106, E45–E46 (2009). 19369203 10.1073/pnas.0813294106 PMC2672534

[b40] Beaumont L. J., Hughes L. & Poulsen M. Predicting species distributions: use of climatic parameters in BIOCLIM and its impact on predictions of species’ current and future distributions. Ecol. Modell. 186, 250–269 (2005).

[b41] Barve N. . The crucial role of the accessible area in ecological niche modeling and species distribution modeling. Ecol. Modell. 222, 1810–1819 (2011).

[b42] Ellis C. J., Coppins B. J., Dawson T. P. & Seaward M. R. D. Response of British lichens to climate change scenarios: Trends and uncertainties in the projected impact for contrasting biogeographic groups. Biol. Cons. 140, 217–235 (2007).

[b43] Ellis C. J. A risk-based model of climate change threat: hazard, exposure, and vulnerability in the ecology of lichen epiphytes. Botany 91, 1–11 (2013).

[b44] Brodo I. M. Studies of the lichen genus *Ochrolechia*. 1. A new classification for *Pertusaria subplicans* and *P. rhodoleuca*. Can. J. Bot. 66, 1264–1269 (1988).

[b45] Boqueras M., Barbero M. & Llimona X. El género *Ochrolechia* A. Massal. (Pertusariaceae, líquenes) en España y Portugal. Cryptogamie Mycol. 20, 303–328 (1999).

[b46] Jia Z.-F. & Zhao Z.-T. A preliminary study of the lichen genus *Ochrolechia* in China. Mycosystema 22, 31–34 (2003).

[b47] Roemer J., Nash T. H. III, Lumbsch H. T. & Messuti M. I. Ochrolechia. In (eds Nash T. H. III, Ryan B. D., Diederich P., Gries C., Bungartz F.) Lichen Flora of the Greater Sonoran Desert Region 2, 381–387 (Lichens Unlimited, Arizona State University, 2004).

[b48] Galloway D. Flora of New Zealand Lichens. Revised Second Edition Including Lichen-Forming and Lichenicolous Fungi. Volumes 1 and 2 (Manaaki Whenua Press, 2007).

[b49] Brodo I. M. Studies in the lichens genus *Ochrolechia*. 2. Corticolous species of North America. Can. J. Bot. 69, 733–772 (1991).

[b50] Parnmen S., Leavitt S. D., Rangsiruji A. & Lumbsch H. T. Identification of species in the *Cladia aggregata* group using DNA barcoding (Ascomycota: Lecanorales). Phytotaxa 115, 1–14 (2013).

[b51] Lücking R. . A single macrolichen constitutes hundreds of unrecognized species. Proc. Natl. Acad. Sci. USA 111, 11091–11096 (2014). 24982168 10.1073/pnas.1403517111 PMC4121827

[b52] Ponce J. F., Rabassa J., Coronato A. & Borromei A. M. Palaeogeographical evolution of the Atlantic coast of Pampa and Patagonia from the last glacial maximum to the Middle Holocene. Biol. J. Linnean Soc. 103, 363–379 (2011).

[b53] Hooghiemstra H. & Ran E. T. H. Late Pliocene-Pleistocene high resolution pollen sequence of Colombia: An overview of climatic change. Quat. Int. 21, 63–80 (1994).

[b54] Baker P. A. Tropical climate changes at millennial and orbital timescales on the Bolivian Altiplano. Nature 409: 698–701 (2001). 11217855 10.1038/35055524

[b55] Seltzer G. . Early warming of the tropical South America at the last glacial-interglacial transition. Science 297, 1685–1686 (2002). 10.1126/science.1070136 12040193

[b56] Abbott M. B. . Holocene paleohydrology and glacial history of the central Andes using multiproxy lake sediment studies. Palaeogeogr. Palaeoclimatol. Palaeoecol. 194, 123–138 (2003).

[b57] Villalba R. . Large-Scale Temperature Changes Across the Southern Andes: 20th-Century Variations in the Context of the Past 400 Years. Clim. Chang. 59, 177–232 (2003).

[b58] Weng C., Bush M. B., Curtis J. H., Kolata A. L., Dillehay T. D. & Binford M. W. Deglaciation and Holocene climate change in the western Peruvian Andes. Quat. Res. 66, 87–96 (2006).

[b59] Vuille M. & Milana J. P. High-latitude forcing of regional aridification along the subtropical west coast of South America. Geophys. Res. Lett. 34, L23703 (2007).

[b60] Rabatel A. . Current state of glaciers in the tropical Andes: a multi-century perspective on glacier evolution and climate change. Cryosphere 7, 81–102 (2013).

[b61] Boulanger J.-P., Martinez F. & Segura E. C. Projection of future climate change conditions using IPCC simulations, neural networks and Bayesian statistics. Part 1: Temperature mean state and seasonal cycle in South America. Clim. Dynam. 27, 233–259 (2006).

[b62] Young K. & León B. Tree-line changes along the Andes: implications of spatial patterns and dynamics. Philos. Trans. R. Soc. Lond. B Biol. Sci. 362, 263–272 (2007). 17255035 10.1098/rstb.2006.1986 PMC2311430

[b63] Orange A., James P. W. & White F. J. Microchemical methods for the identification of lichens (British Lichen Society, 2001).

[b64] Phillips S. J., Dudík M. & Schapire R. E. A maximum entropy approach to species distribution modeling. in ICML ′04 Proceedings of the twenty-first international conference on Machine learning, 655–662 (ACM, 2004).

[b65] Elith J., Phillips S. J., Hastie T., Dudík M., Chee Y. E. & Yates C. J. A statistical explanation of MaxEnt for ecologists. Divers. Distrib. 17, 43–57 (2011).

[b66] Pearson R. G., Raxworthy C. J., Nakamura M. & Peterson A. T. Predicting species distributions from small numbers of occurrence records: a test case using cryptic geckos in Madagascar. J. Biogeogr. 34, 102–117 (2006).

[b67] Wisz M. S. . Effects of sample size on the performance of species distribution models. Divers. Distrib. 14, 763–773 (2008).

[b68] Hijmans R. J., Cameron S. E., Parra J. L., Jones P. G. & Jarvis A. Very high resolution interpolated climate surfaces for global land areas. Int. J. Climatol. 25, 1965–1978 (2005).

[b69] Urbina-Cardona J. N. & Loyola R. D. Applying niche-based models to predict endangered-hylid potential distributions: are neotropical protected areas effective enough? Trop. Conserv. Sci. 1, 417–445 (2008).

[b70] Braconnot P. . Results of PMIP2 coupled simulations of the mid-Holocene and Last Glacial Maximum, Part 1: experiments and large-scale features. Clim. Past 3, 261–277 (2007).

[b71] Mitchell T. D. & Osborn T. J. *ClimGen: a flexible tool for generating monthly climate data sets and scenarios* (Tyndall Centre for Climate Change Research Working Paper, 2005).

[b72] Ramirez J. & Jarvis A. *High resolution statistically downscaled future climate surfaces* (International Centre for Tropical Agriculture, CIAT, 2008).

[b73] Stockwell D. R. B. & Peters D. G. The GARP modelling system: Problems and solutions to automated spatial prediction. International Journal of Geographic Information Systems 13, 143–158 (1999).

[b74] Muñoz M. E. S. . OpenModeller: a generic approach to species’ potential distribution modelling”. GeoInformatica 15, 111–135 (2011).

[b75] Schoener T. W. The anolis lizards of bimini: Resource partitioning in a complex fauna. Ecology 49, 704–726 (1968).

[b76] Warren D. L., Glor R. E. & Turelli M. Environmental niche equivalency versus conservatism: Quantitative approaches to niche evolution. Evolution 62, 2868–2883 (2008). 18752605 10.1111/j.1558-5646.2008.00482.x

[b77] Warren D. L., Glor R. E. & Turelli M. ENMTools: a toolbox for comparative studies of environmental niche models. Ecography 33, 607–611 (2010).

[b78] ESRI. *ArcGIS 9.2* (Environmental Systems Research Institute Inc., 2006).

[b79] QGIS Development Team. QGIS Geographic Information System. Open Source Geospatial Foundation Project. http://qgis.osgeo.org (2016).

