# Supplementary material for: Glacial refugia and the prediction of future habitat coverage of the South American lichen species Ochrolechia austroamericana
Source: Sci Rep. 2016 Dec 8;6:38779. doi: 10.1038/srep38779 (PMC5144090; doi:10.1038/srep38779)
Supplement: Supplementary Dataset 1 [file srep38779-s1.doc]

**Glacial refugia and the prediction of future habitat coverage of the South American lichen species *Ochrolechia austroamericana***

Martin Kukwa and Marta Kolanowska

**Supplementary Table S1.** Reliability of models created using GARP.

|  | **Present** | **A1** | **A2** | **B** |
| --- | --- | --- | --- | --- |
| **AUC** | 0.98 | 0.98 | 0.97 | 0.98 |
| **Sensivity** | 0.711 | 0.650 | 0.675 | 0.825 |

**Supplementary Figure S2.** Current distribution of ecological niches of *O. austroamericana* based on “SelLay” (a) and “SelArea” (b) models. Maps generated in ArcGis 9.2^80^ (http://www.esri.com/).


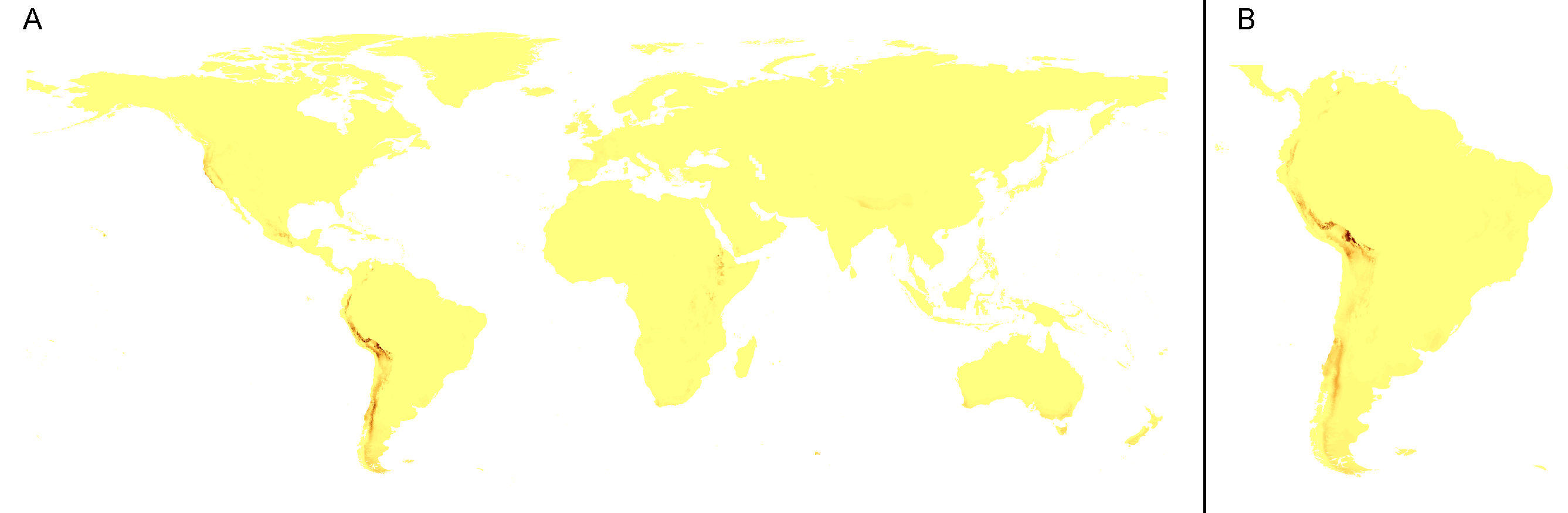


**Supplementary Figure S3.** Distribution of ecological niches of *O. austroamericana* during LGM based on “SelLay” (a) and “SelArea” (b) models. Maps generated in ArcGis 9.2^80^ (http://www.esri.com/).


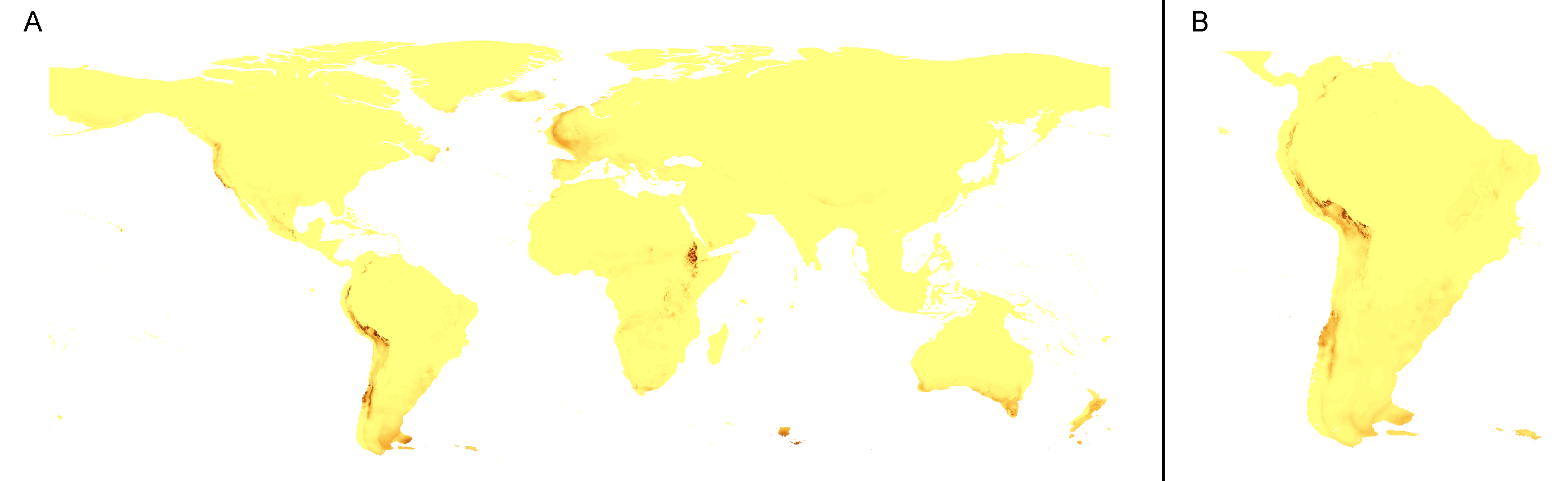


**Supplementary Figure S4.** Predicted South American distribution of ecological niches of *O. austroamericana* in 2080. “SelLay” models based on A1b (a) A2a (b) and B2a (c) climate changes scenarios. “SelArea” models based on A1b (d) A2a (e) and B2a (f) climate changes scenarios. Maps generated in ArcGis 9.2^80^ (http://www.esri.com/).


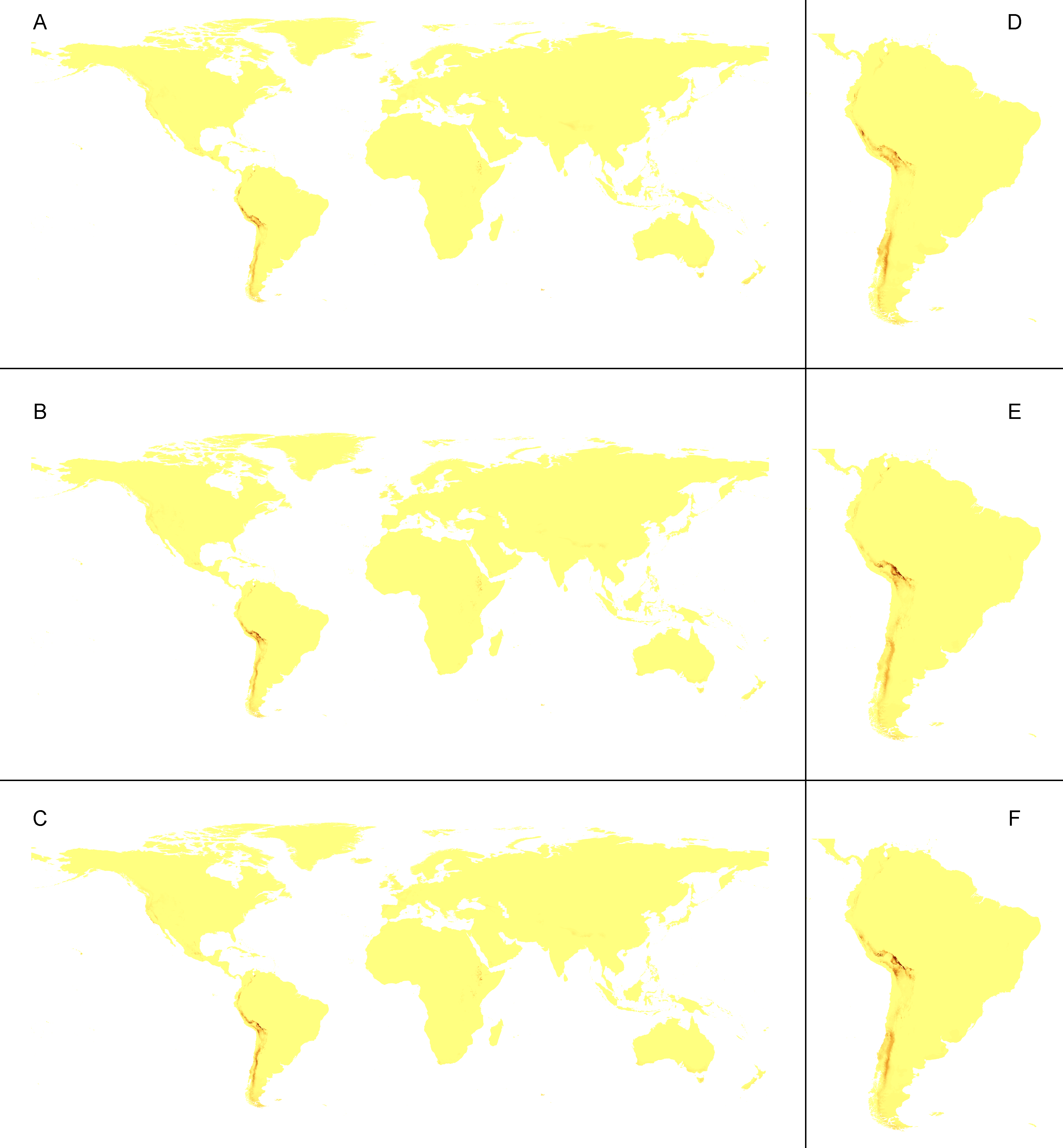


**Supplementary Figure S5.** Models generated using GARP algorithm for present time (A), future A1b (B), A2a (C), and B2a (D) scenarios. Maps generated in ArcGis 9.2^80^ (http://www.esri.com/).


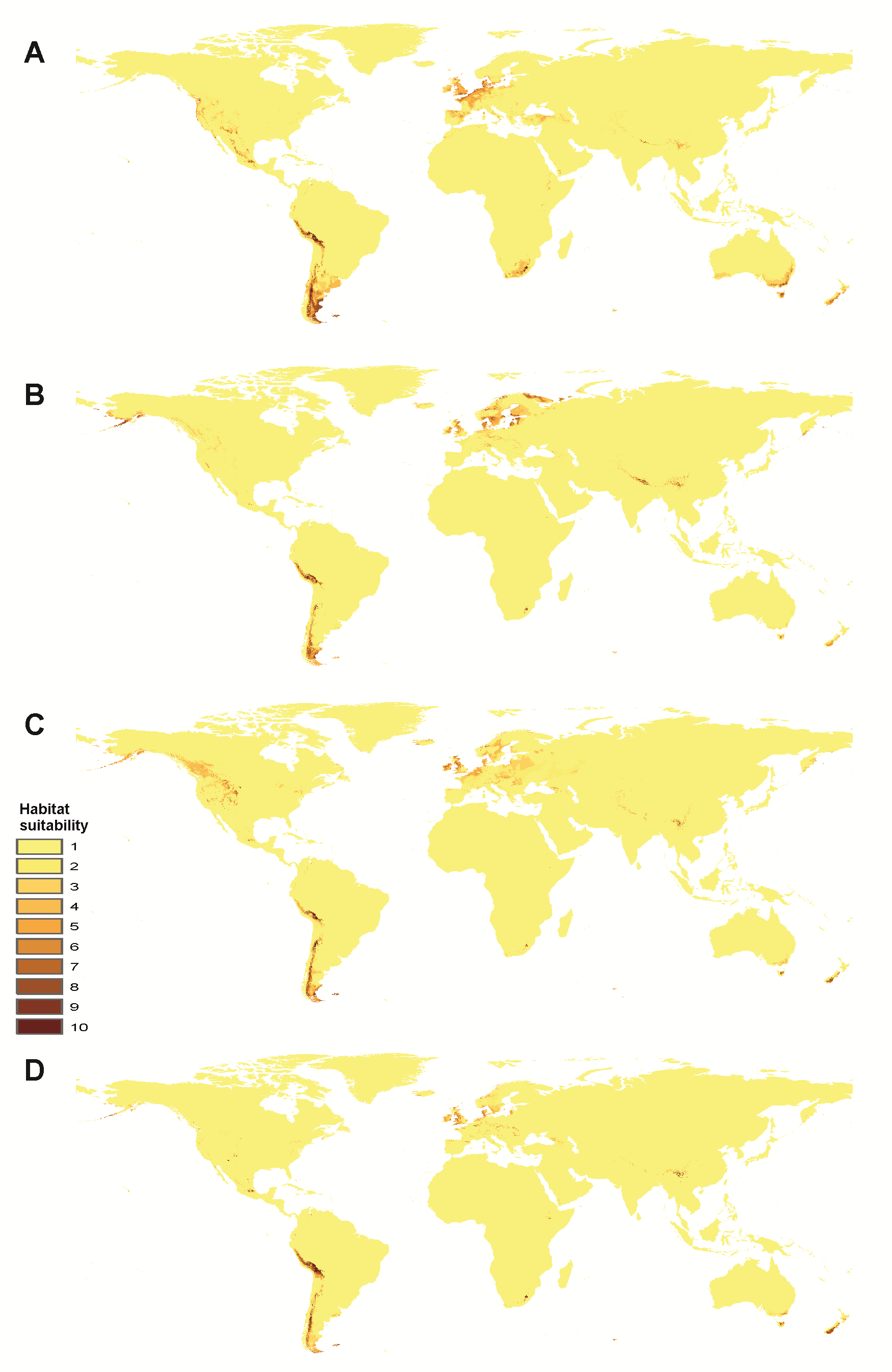


**Supplementary Table S6.** World-wide coverage of areas characterized by bioclimatic conditions of various suitability for *O. austroamericana* in present time and in various climate changes scenarios calculated based on GARP models.

| **Suitability** | **Present time** | **A1b** | **A2a** | **B2a** |
| --- | --- | --- | --- | --- |
| > 0.8 | 116077.78 | 16452.82 | 64643.8 | 129957.8 |
| 0.7-0.8 | 218210.66 | 87885.3 | 143362.2 | 239138.8 |
| > 0.7 | 654459.02 | 305339.3 | 298961.4 | 390478.8 |

**Supplementary Table S7.** List of localities of *Ochrolechia austroamericana* used in the modeling.

| **Location** | **Coordinates** | **Collector** | **Nr** | **Reference** |
| --- | --- | --- | --- | --- |
| Chile, V Region: Parque Nacional La Campana, alt. 1000-1400 m, near Darwin Monument. | -71,1333; -32,9333 | B. J. Coppins, D. J. Galloway, G. Guzmán, P. W. James | 5570 | BM |
| Chile, Biobío Region, Chillán, Inveruade, Inveruade (CONC 277d). | -72,1144; -36,6161 | *Sine coll.* |  | BM |
| Chile, M-Chile, Prov. Bio-Bio, Anden, Umgebung des Refugio an der Laguna de Laja, alt. ± 1600 m. | -71,3; -37,3167 | Rorgiguez, J. Poelt |  | GZU |
| Ecuador, prov. Pichincha, Guamaní (páramo de la Virgen), a moist páramo N of road (Quito) Pifo - Papallacta, ca 25 km WSW of Quito, alt. 4200 m. | -78,2; -0,3 | Z. Palice | 2280 | PRA |
| Argentina, [Argentina, Patagonia, Prov. Neuquén] Parque de N. H. [Nahuel Huapí], Valle Limay, 5 km austr. Lipela, en rocas, alt. ca. 750 m. | -71,1186; -40,8419 | A. Donat |  | H |
| Uruguay, Montevideo, Cerro, alt. 50-100 m. | -56,2536; -32,8825 | W. G. Herter |  | H |
| Bolivia, Dept. Oruro, Prov. Sajama, Parque Nacional Sajama, near Sajama village, alt. 4437 m, Puna sureña, Tholares vegetation, | -68,9483; -18,1303 | A. Flakus, P. Rodriquez | 16455 | KRAM-L– |
| Bolivia, Dept. Oruro, Prov. Sajama, Parque Nacional Sajama, Huincurata near Sajama village, alt. 4301 m, Puna sureña, Polylepis vegetation (Queñuales). | -68,9667; -18,1169 | A. Flakus, P. Rodriquez | 16569 | KRAM-L– |
| Bolivia, Dept. Oruro, Prov. Sajama, Parque Nacional Sajama, Jecha K’ala 25 km of Sajama village, alt. 4184 m, Puna sureña, Pajonales vegetation. | -68,8189; -18,1644 | A. Flakus, P. Rodriquez | 16634 | KRAM-L– |
| Bolivia, Dept. La Paz, Prov. Bautista Saavedra, Area Natural de Manejo Integrado Nacional Apolobamba, near Villa Amarca village, alt. 4643 m, Puna Húmeda vegetation. | -69,0297; -15,2797 | A. Flakus, P. Rodriquez | 17367 | KRAM-L– |
| Bolivia, Dept. La Paz, Prov. Franz Tamayo, Area Natural de Manejo Integrado Nacional Apolobamba, Socondori Chico near Ulla Ulla village, alt. 4479 m, high Andean open vegetation. | -69,23; -15,0106 | A. Flakus, P. Rodriquez | 17440 | KRAM-L– |
| Bolivia, Dept. La Paz, Prov. Franz Tamayo, Area Natural de Manejo Integrado Nacional Apolobamba, near Puyo Puyo village, alt. 4888 m, high Andean open vegetation. | -69,1328; -15,9486 | A. Flakus, P. Rodriquez | 17592 | KRAM-L– |
| Bolivia, Dept. La Paz, Prov. Camacho, Pacoamba cerca Wila Kala, alt. 4283 m, Puna Húmeda vegetation. | -69,0111; -15,4111 | A. Flakus, P. Rodriquez | 17698 | KRAM-L– |
| Bolivia, Dept. La Paz, Prov. Omasuyos, El Dragon hill cerca Chahualla, alt. 3850 m, Puna Húmeda vegetation. | -69,0111; -15,8547 | A. Flakus, P. Rodriquez | 17839 | KRAM-L– |
| Bolivia, Dept. La Paz, Prov. Nor Yungas, near Pongo village, near the road between Coroico and La Paz, 3820 m. | -67,9558; -16,3244 | M. Kukwa | 9407 | UGDA-L– |
| Bolivia, Dept. La Paz, Prov. Murillo, below Potosí, on the road between La Paz and Valle del Zongo, alt. 4720 m. | -68,1283; -16,2953 | M. Kukwa | 9493 | UGDA-L– |
| Bolivia, Dept. La Paz, Prov. Franz Tamayo, Cerca del campamento de guardaparques del PNANMI Apolobamba, Puntani, alt. 4770 m. | -69,1856; -15,0306 | P. Rodriguez | 528 | LPB |
| Bolivia, Dept. La Paz, Prov. Franz Tamayo, A dos horas de caminanta desde la comunidad de Puyo Puyo, cerro Pelechucomita, alt. 5050 m. | -69,1467; -15,02167 | P. Rodriguez | 554 | LPB |
| Bolivia, Dept. La Paz, Prov. Murillo, Parque Nacional Tuni - Condoriri, Comunidad Tuni, a 15 minutos de la cabaña de la represa Tuni. Cima Condor Pustaña, alt. 4862 m. | -68,2675; -16,2242 | P. Rodriguez | 974 | LPB |
| Bolivia, Dept. La Paz, Prov. Camacho cerca Villa Cala village, alt. 4250 m. | -69,0853; -15,4314 | A. Flakus | 21197 | KRAM-L– |
| Bolivia, Dept. La Paz, Prov. Manco Kapac, near Copacabana village Mt. Horca del Inca, alt. 3974 m. | -69,0847; -16,1708 | A. Flakus | 8669 | KRAM-L– |
| Argentina, Prov. Mendoza, Las Heras, Portezuelo de las Avispas. | -69,1208; -33,0717 | Ruiz Leal | 6005 | Messuti & Lumbsch 2000 (Bibl. Lichenol.) |
| Argentina, Prov. Neuquén, Rincón Grande, near Río Limay. | -70,1572; -50,2919 | Messutti | 310 | Messuti & Lumbsch 2000 (Bibl. Lichenol.) |
| Chile, V Region: Parque Nacional La Campana, alt. 1200 m, near Darwin Monument. | -71,1333; -32,9333 | B. J. Coppins, D. J. Galloway, G. Guzmán, P. W. James | 5604 | BM |
| Chile, M-Chile, Prov. Bio-Bio, Anden, Umgebung des Refugio an der Laguna de Laja. | -71,3; -37,3167 | Rorgiguez, J. Poelt |  | GZU |
| Chile, V Region, Parque Nacional La Campana, alt. 1000-1400 m. | -71,1333; -32,9333 | B. J. Coppins, D. J. Galloway, G. Guzman, P. W. James | 5570 | BM |
| Bolivia, Dept. Oruro, Prov. Sajama, Parque Nacional Sajama, near Sajama village, alt. 4437 m, Puna sureña, Tholares vegetation. | -68,9483; -18,1303 | A. Flakus, P. Rodriquez | 16502 | KRAM-L– |
| Bolivia, Dept. Oruro, Prov. Sajama, Parque Nacional Sajama, near Sajama village, alt. 4437 m, Puna sureña, Tholares vegetation. | -68,9483; -18,1303 | A. Flakus, P. Rodriquez | 16504 | KRAM-L– |
| Bolivia, Dept. Oruro, Prov. Sajama, Parque Nacional Sajama, Huincurata near Sajama village, alt. 4301 m, Puna sureña, Polylepis vegetation (Queñuales). | -68,9667; -18,1169 | A. Flakus, P. Rodriquez | 16538 | KRAM-L– |
| Bolivia, Dept. Oruro, Prov. Sajama, Parque Nacional Sajama, Huincurata near Sajama village, alt. 4301 m, Puna sureña, Polylepis vegetation (Queñuales). | -68,9667; -18,1169 | A. Flakus, P. Rodriquez | 16538 | UGDA-L– |
| Bolivia, Dept. Oruro, Prov. Sajama, Parque Nacional Sajama, Huincurata near Sajama village, alt. 4301 m, Puna sureña, Polylepis vegetation (Queñuales). | -68,9667; -18,1169 | A. Flakus, P. Rodriquez | 16561 | KRAM-L– |
| Bolivia, Dept. Oruro, Prov. Sajama, Parque Nacional Sajama, Jecha K’ala 25 km of Sajama village, alt. 4184 m, Puna sureña, Pajonales vegetation. | -68,8189; -18,1644 | A. Flakus, P. Rodriquez | 16654 | KRAM-L– |
| Bolivia, Dept. Oruro, Prov. Sajama, Parque Nacional Sajama, Jecha K’ala 25 km of Sajama village, alt. 4184 m, Puna sureña, Pajonales vegetation. | -68,8189; -18,1644 | A. Flakus, P. Rodriquez | 16705 | KRAM-L– |
| Bolivia, Dept. La Paz, Prov. Bautista Saavedra, Area Natural de Manejo Integrado Nacional Apolobamba, near Villa Amarca village, alt. 4643 m, Puna Húmeda vegetation. | -69,0297; -15,2797 | A. Flakus, P. Rodriquez | 17379 | KRAM-L– |
| Bolivia, Dept. La Paz, Prov. Franz Tamayo, Area Natural de Manejo Integrado Nacional Apolobamba, Socondori Chico near Ulla Ulla village, alt. 4479 m, high Andean open vegetation. | -69,23; -15,0106 | A. Flakus, P. Rodriquez | 17442 | KRAM-L– |
| Bolivia, Dept. La Paz, Prov. Franz Tamayo, Area Natural de Manejo Integrado Nacional Apolobamba, Socondori Chico near Ulla Ulla village, alt. 4479 m, high Andean open vegetation. | -69,23; -15,0106 | A. Flakus, P. Rodriquez | 17450 | KRAM-L– |
| Bolivia, Dept. La Paz, Prov. Franz Tamayo, Area Natural de Manejo Integrado Nacional Apolobamba, Socondori Chico near Ulla Ulla village, alt. 4479 m, high Andean open vegetation. | -69,23; -15,0106 | A. Flakus, P. Rodriquez | 17455 | KRAM-L– |
| Bolivia, Dept. La Paz, Prov. Camacho, Pacoamba cerca Wila Kala, alt. 4283 m, Puna Húmeda vegetation. | -69,0733; -15,4111 | A. Flakus, P. Rodriquez | 17698 | UGDA-L– |
| Bolivia, Dept. La Paz, Prov. Camacho, Pacoamba cerca Wila Kala, alt. 4283 m, Puna Húmeda vegetation. | -69,0733; -15,4111 | A. Flakus, P. Rodriquez | 17706 | KRAM-L– |
| Bolivia, Dept. La Paz, Prov. Camacho, Pacoamba cerca Wila Kala, alt. 4283 m, Puna Húmeda vegetation. | -69,07333; -15,4111 | A. Flakus, P. Rodriquez | 17737 | KRAM-L– |
| Bolivia, Dept. La Paz, Prov. Camacho, Pacoamba cerca Wila Kala, alt. 4283 m, Puna Húmeda vegetation. | -69,0733; -15,4111 | A. Flakus, P. Rodriquez | 17777 | KRAM-L– |
| Bolivia, Dept. La Paz, Prov. Murillo, Parque Nacional Tuni - Condoriri, Comunidad Tuni, a 20 minutos de la cabaña de la represa Tuni y dos horas de caminata. Cima Paco Thojo. 5014 m. | -68,27; -16,2086 | P. Rodriguez | 1030 | LPB |
| Bolivia, Dept. La Paz, Prov. Murillo, Parque Nacional Tuni - Condoriri, Comunidad Tuni, a 20 minutos de la cabaña de la represa Tuni y dos horas de caminata. Cima Paco Thojo, alt. 5014 m. | -68,27; -16,2086 | P. Rodriguez | 1038 | LPB |
| Bolivia, Dept. La Paz, Prov. Murillo, Parque Nacional Tuni - Condoriri, Comunidad Tuni, a 20 minutos de la cabaña de la represa Tuni y dos horas de caminata. Cima Paco Thojo, alt. 5014 m. | -68,2083; -16,2086 | P. Rodriguez | 1056 | LPB |
| Bolivia, Dept. La Paz, Prov. Murillo, below Potosí, on the road between La Paz and Valle del Zongo. | -68,1283; -16,2953 | A. Flakus | 21880 | KRAM-L– |
